# Brain Responses Underlying Anthropomorphism, Agency, and Social Attribution in Autism Spectrum Disorder

**DOI:** 10.2174/1874440001812010016

**Published:** 2018-03-30

**Authors:** Carla J. Ammons, Constance F. Doss, David Bala, Rajesh K. Kana

**Affiliations:** Department of Psychology, University of Alabama at Birmingham, Birmingham, AL, USA

**Keywords:** Autism, Animation, Anthropomorphism, Theory of Mind, Social attribution, Functional MRI, Social brain

## Abstract

**Background:**

Theory of Mind (ToM), the ability to attribute mental states to oneself and others, is frequently impaired in Autism Spectrum Disorder (ASD) and may result from altered activation of social brain regions. Conversely, Typically Developing (TD) individuals overextend ToM and show a strong tendency to anthropomorphize and interpret biological motion in the environment. Less is known about how the degree of anthropomorphism influences intentional attribution and engagement of the social brain in ASD.

**Objective:**

This fMRI study examines the extent of anthropomorphism, its role in social attribution, and the underlying neural responses in ASD and TD using a series of human stick figures and geometrical shapes.

**Methods:**

14 ASD and 14 TD adults watched videos of stick figures and triangles interacting in random or socially meaningful ways while in an fMRI scanner. In addition, they completed out-of-scanner measures of ToM skill and real-world social deficits. Whole brain statistical analysis was performed for regression and within and between group comparisons of all conditions using SPM12’s implementation of the general linear model.

**Results:**

ToM network regions were activated in response to social movement and human-like characters in ASD and TD. In addition, greater ToM ability was associated with increased TPJ and MPFC activity while watching stick figures; whereas more severe social symptoms were associated with reduced right TPJ activation in response to social movement.

**Conclusion:**

These results suggest that degree of anthropomorphism does not differentially affect social attribution in ASD and highlights the importance of TPJ in ToM and social attribution.

## 1 INTRODUCTION

The ability to understand and predict the behavior of other agents, who possess their own thoughts, desires, goals, and intentions, agents whose motives are often hidden and must be inferred from their actions, is one of the most complex cognitive tasks the human brain has to accomplish [1]. It is also one with enormous evolutionary benefit [2, 3]. Rapidly detecting the movements of agents in the social world allows for speedy threat detection and accurate prediction of behavior. This ability relies on two processes, identification of biological movement in the environment and attribution of intentionality to the entity engaged in that movement. Both processes impact adaptive social behavior including day-to-day social interaction and nonverbal communication [4].

In Social Psychology, the term Theory of Mind (ToM) is used to describe the ability to attribute mental states to oneself and others. The ToM account of Autism Spectrum Disorder (ASD) attributes the social communication deficits in ASD to a dysfunctional ToM system [5, 6]. While this model may not explain the entire spectrum of autism symptoms, deficits in ToM have been consistently documented and linked to real-life social difficulties [6, 7]. The development of basic ToM skills in children with ASD is often delayed compared to mental age-matched peers and to the development of their other cognitive skills [8, 9]. Furthermore, precursors to ToM including joint-attention, gaze following, and pretend play are often absent, limited, or delayed in infants later diagnosed with autism [10-13]. High functioning adolescents and adults with ASD, despite demonstrating substantial real world social difficulties, are often able to pass commonly used laboratory tasks of ToM [14-16]. It is noteworthy that performance on traditional ToM tasks correlates with verbal IQ in typically developing and patient populations [8, 17]. Verbal reasoning, therefore, may help inflate performance. Another aspect of traditional ToM tasks is the direct cueing of attention to others’ thoughts, intentions, or emotions since many tasks explicitly ask what someone is thinking or highlight relevant social cues in context [15]. This explicit cueing may provide an advantage that is not present in real-world social settings, thus not capturing the true social difficulties of the higher functioning population.

In order to address the limitations of traditional ToM tasks, the social attribution task was adapted from a classic study demonstrating that the movement of simple geometric shapes gives rise to an impression of intentional action and provokes the attribution of complex mental states [18]. In this task, shapes may be perceived as biological agents with beliefs, desires, and intention. Typically developing (TD) individuals identify the social nature of the movement relatively quickly and provide descriptions that include social motives (*i.e.* protecting, bullying), relationships between the characters (*i.e.* mother and child, friends), and attribution of mental states (*i.e.* scared, shy). In contrast, individuals with ASD, while accurately describing mechanical or goal directed movements of the shapes, did not pick up on the social or relational aspects of the shape movement or tend to use mental state language to describe the action despite being able to pass other higher order ToM tasks [19-21]. This suggests that even in high functioning individuals with ASD, spontaneous attribution of intentions to events in the social world may be problematic and the social attribution task is sensitive to subtle social cognition deficits.

Neuroimaging studies across different ToM tasks have identified a common network of brain regions that show increased activity. This includes the Medial Prefrontal Cortex (MPFC), the posterior superior temporal sulcus (pSTS) at the Temporal-Parietal Junction (TPJ), and the temporal pole anterior to the amygdala [22-24]. These regions are consistently activated when making inferences about others’ mental states, regardless of the specific task demands (*i.e.* false belief tasks, verbally-based mentalizing tasks, picture completion or comic strip vignettes, and animated shape paradigms) suggesting an underlying, integrated network. Studies in ASD have shown hypoactivation of these ToM regions, even in the presence of similar behavioral performance [25-27]. Interestingly, many of these same regions are involved in the detection of biological motion, particularly the right STS [28, 29]. It has been proposed that biological motion processing is closely associated with the development of social cognitive abilities [29] and individuals with ASD have also demonstrated deficits in biological motion processing [30-32]. This includes difficulty detecting camouflaged biological motion [33] and discriminating biological motion in point light displays [34].

Three previous studies have examined the neural responses underlying the attribution of mental states to moving shapes in ASD, a task that combines ToM, anthropomorphism, and biological motion processing. In Castelli *et al.*’s [25] study, high functioning adults with ASD activated the MPFC, STS, and temporal pole areas to a lesser extent than well-matched control participants when viewing shapes moving interactively with social motives. Furthermore, extrastriate regions which activated strongly during the mentalizing condition for both groups showed reduced connectivity to the STS in the ASD group [25] suggesting reduced communication between these regions. In two studies from Kana and colleagues, high functioning adults with ASD demonstrated reduced activation in ToM and mirror network regions, including MPFC, TPJ, and cingulate cortex, when viewing social animations as well as reduced connectivity between frontal and posterior ToM regions [26, 27]. Furthermore, activation in the right TPJ was correlated with ToM ability in the ASD group [27]. These studies suggest that regions of the ToM network are underactive and underconnected in ASD when making spontaneous social attributions.

What is less understood is how specific characteristics of the agents performing the action influence social attribution and anthropomorphism in ASD. The tendency toward anthropomorphism is well-documented in TD individuals; where attributing human-like beliefs, intentions, and emotions to animals and even inanimate objects is common. Motivational aspects as well as cognitive mechanisms have been proposed to explain anthropomorphic tendency with some considering anthropomorphism the brain’s “default” processing style in the face of ambiguous input [35]. Other cognitive mechanisms including attribution of agency, biological motion detection, motor simulations and the mirror neuron system, embodied cognition, empathy, and causal reasoning have all been proposed as contributors toward anthropomorphic bias [35]. Many of these processes have also been implicated in social cognition difficulties in ASD and are building blocks in the social attribution process. However, the nature and characteristics of the agent of action can influence anthropomorphism as a response bias. For example, Chaminade *et al.* [36] showed that motion performed by more anthropomorphic computer generated characters reduces bias toward interpreting movement as biological, the “uncanny valley” hypothesis. At the same time, response bias towards perceiving motion as biological, regardless of character, was associated with increased activation of left STS, left TPJ, right Superior Temporal Gyrus (STG), Anterior Cingulate Cortex (ACC), and precuneus – regions considered to be crucial in processing ToM [36]. A follow-up study showed that children with ASD did not display the characteristic anthropomorphic bias of TD children [37]. Instead, children with ASD were not influenced by the degree of congruence between anthropomorphism of the character and movement suggesting they failed to integrate context and/or recognize the social information in the character. Interestingly, anthropomorphism bias has not been studied in the context of the social attribution task in ASD.

The present fMRI study examines the extent of anthropomorphism, its role in social attribution, and the underlying neural responses in ASD and TD using a series of stick figure human characters and geometrical shapes. We investigate the impact of an inherently social character (a stick figure human) on perceived biological motion, social attribution, and brain activity underlying both. We presented short video clips of moving two-dimensional characters and asked participants to identify whether or not the characters were moving in an intentional, socially directed manner. Importantly, we manipulated aspects of the agent’s movements (social *vs*. nonsocial) as well as aspects of the agents themselves (social *vs* nonsocial) in order to test the differential contributions of motion processing and character features (geometrical shapes *vs.* humanlike stick figures) to agency and social attribution. This study is novel in that it examined both movement and agent characteristics affecting social attribution in the same well-defined sample of adults with ASD compared to age-and-IQ-matched peers. Previous research has focused almost exclusively on the movement of the characters rather than the characters themselves. As such, this study is unique in that it can comment on the impact of social aspects of the characters as well as their movement and how this differs between ASD and TD participants. Based on previous studies, we hypothesized that participants with ASD would have more difficulty detecting social motives in the movements of both humanlike and shape characters and that this difficulty would be reflected in reduced activation of key ToM regions (MPFC, pSTS, TPJ) during social attribution regardless of character. We also hypothesized that viewing more anthropomorphic characters, regardless of the type of movement they were performing, would more strongly activate social brain regions and that this would be most apparent in the TD group due to the inherent social/biological cue of the stick figure. Lastly, we expected that ToM network activity during social attribution based on movement or character information would predict general ToM abilities measured outside the scanner.

## 2 METHODS

### 2.1 Participants

Functional MRI data were collected from 17 TD young adults and 17 high functioning adults with ASD. Data from three individuals in the ASD group were excluded due to equipment failure (1) and inattentive responding (2), defined as an in-window response to less than 50% of stimuli or accuracy below 65%. Three individuals from the TD group were excluded due to excessive head motion (1), equipment failure (1), and inattentive responding (1). This resulted in a final sample of 14 TD and 14 ASD participants (Table **1**). Groups were matched on age, IQ, and handedness. Average head motion, defined as the root mean square of displacement, did not differ between ASD and TD groups (t(28,2)=1.47, p=0.15) and all participants retained greater than 80% of functional volumes after censoring.

Participants with ASD were recruited from local service providers in the Birmingham and Tuscaloosa areas including the UAB Civitan-Sparks clinic, Mitchell’s Place, the Glenwood Foundation, and the University of Alabama ASD Clinic, as well as from previous involvement with the Cognition, Brain, and Autism Lab at UAB. Previous ASD diagnosis *via* the Autism Diagnostic Observation Schedule (ADOS) and/or the Autism Diagnostic Interview (ADI) was required and verified by patient records. TD participants were recruited from the UAB campus and the Birmingham area at large. All participants were screened for and excluded based on claustrophobia; body mass index exceeding 34; history of working with metal or non-removal metallic body implants; medical conditions affecting blood flow including diabetes, hypertension, sickle cell disease, and anemia; current use of psychotropic medications; and any reported history of neurological disorder. Prior to scanning, all study procedures were described to participants and informed consent was obtained. All aspects of this study were approved by the UAB Institutional Review Board.

### 2.2 Behavioral Measures

All participants completed the following measures outside of the scanner: Wechsler Abbreviated Scale of Intelligence (WASI), Ritvo Autism Asperger Diagnostic Scale-Revised (RAADS-R), Reading the Mind in the Eyes (RMIE) test, and the Edinburgh Handedness Inventory. Due to task demands, inclusion in the study required a full-scale IQ estimate of 80 or higher on the WASI. The RAADS-R is a self-report measure of Autism symptomology in adults used in clinical diagnosis [38]. Of interest to the current study is the Social Relatedness scale, which taps into empathy, quality of relationships, and social language. RMIE is an advanced theory of mind task and involves deciphering mental or emotional states from photos of eyes [39].

### 2.3 Stimuli and Experimental Design

**Fig. (1):**
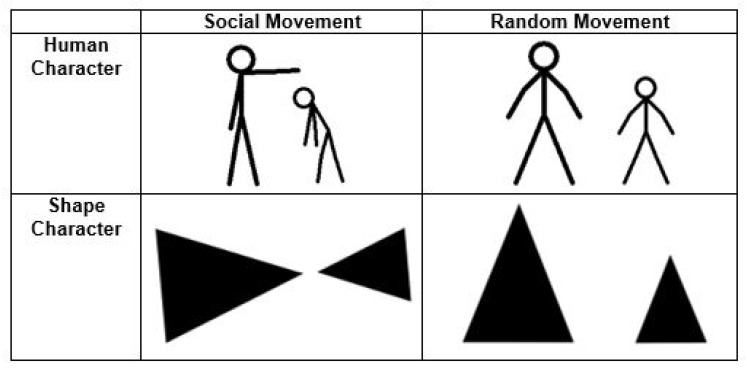
Example static images of the dynamic experimental stimuli depicting socially oriented and random movement performed by human-like stick figures and triangle characters.

The stimuli consisted of 20 short black and white video clips adapted and expanded from Hieder and Simmel’s original animations [18]. Each clip lasted between 9-21 seconds and depicted either two triangles or two stick figure human characters interacting (Fig. **1**). In each video, the characters performed either socially oriented (*i.e.* bullying, helping) or random (*i.e.* geometric patterns) actions. Participants were asked to determine whether the character’s actions were socially motivated or random. All stimuli were piloted on a separate group of TD adults to ensure that the animations reliably distinguished between social and random action. Only those items with greater than 80% agreement were retained.

The stimuli were presented in an event related design interspersed with a 24 second fixation interval. Each video was viewed once over the course of the experiment and the order of presentation was pseudorandomized. Brief instructions were given on the screen prior to each animation and participants indicated their response (social or random) *via* button press. Reaction time and performance data were obtained from these behavioral responses. All participants practiced the task outside of the scanner prior to scan and animations used for practice were not repeated in the experiment. Inquisit 2 (Millisecond Software, Seattle WA, USA) software was used to present the video clips in the scanner *via* projection onto a screen that was viewed by the participant using a mirror.

### 2.4 MRI Data Acquisition

Structural and functional MRI data were acquired on a 3T Siemens Allegra head-only scanner (Siemens Medical Inc., Erlangen, Germany) located in the Civitan International Research Center at UAB. High resolution T1-weighted scans were acquired using a 160 slice 3D magnetization prepared rapid gradient echo (MPRAGE) volume scan with repetition time (TR) = 200 ms, echo time (TE) = 3.34 ms, flip angle = 12 degrees, field of view (FOV) = 25.6cm, matrix size = 256x256, and slick thickness = 1 mm. Functional T2* images were acquired using a single-shot gradient-recalled echo-planner pulse sequence with TR = 1000 ms, TE = 30 ms, flip angle = 60 degrees, FOV = 24 cm, matrix = 64x64. This allowed for rapid image acquisition with sufficient coverage of the brain *via* seventeen 5mm thick oblique-axial slices with a 1mm gap obtained in interleaved sequence. The resulting in-plane resolution was 3.75mm x 3.75mm x 5mm and the relatively thicker slices allowed higher signal to noise ratio.

### 2.5 Data Preprocessing and Analysis

Structural and functional images were preprocessed and analyzed using Statistical Parametric Mapping (SPM12) software (Wellcome Department of Imaging Neuroscience, London, UK). Functional images were slice-time corrected, head motion corrected *via* registration of each functional volume to the middle time point of the scan, resampled to 2 x 2 x 2 mm^3^ isotropic voxels, and smoothed using a 6mm Gaussian kernel to reduce spatial noise. These images were then coregistered to the high resolution 3D anatomical image and warped to a standard atlas space using deformation fields to realign the subject specific 3D anatomical image into MNI space for group comparisons.

### 2.6 Statistical Analyses

Whole-brain statistical analyses were performed at an individual and group level using SPM12’s implementation of the general linear model. For first level analysis, linear regression was performed at each voxel using generalized least squares with a global approximate AR (1) autocorrelations model, drift fit with discrete cosine transform basis (128s cut-off). Second level analysis also utilized voxel-wise linear regression using ordinary least squares. Separate regressors were modeled for each experimental condition (social human, social shape, random human, and random shape) and fixation. Six rigid-body motion parameters corresponding to head motion correction were included as covariates. The following orthogonal contrasts were calculated for within and between group analyses: Social *vs* Random Movement, Human vs Shape Character, and each condition over fixation to ensure task fidelity. One and two sample t-tests were conducted to determine areas of significant activation within each contrast.

In order to control for alpha inflation resulting from the large number of statistical tests used in the massive univariate approach, Monte Carlo simulations were performed *via* AFNI’s 3dClusSim [40] to determine an appropriate cluster-wise correction threshold equivalent to a family-wise error corrected threshold of p < 0.05. Based on this simulation an uncorrected threshold of p < 0.005 with cluster extent threshold greater than 80 contiguous voxels was used. Finally, activation results were entered into regression analysis with behavioral measures of interest including RMIE and RAADS-R social responsiveness.

## 3 RESULTS

### 3.1 Overview

The main results of this study are: 1) TD and ASD participants were equally accurate at detecting social movement of geometric and human like shapes; 2) Observation of social movement, regardless of the type of agent (human figure or geometrical shape), recruited bilateral ToM regions, including the pSTS, TPJ, and MPFC; 3) Direct comparison between groups revealed greater activity in regions primarily associated with visual processing (lingual gyrus, calcarine sulcus) during social movement for TD, but increased medial frontal activity for human-like characters in ASD; 4) Better ToM skills in the participants predicted greater activation in bilateral TPJ and MPFC when watching human-like characters; and 5) More severe ASD symptoms were associated with reduced activity in the RTPJ while observing socially oriented movement.

### 3.2 Behavioral Data Analysis

As expected, participants with ASD reported greater autism symptoms (t(28,2)=8.30, p<.001) and displayed poorer performance on an advanced ToM measure (t(28,2)=2.01, p=.05). On the in-scanner ToM task, independent samples t-tests revealed no significant group differences in performance accuracy or reaction time for any of the four combinations of character (shape, human) and movement (social, random) types (Table **2**).

### 3.3 Brain Activation – Within and Between Groups Analysis

#### 3.3.1 Social Movement

**Fig. (2):**
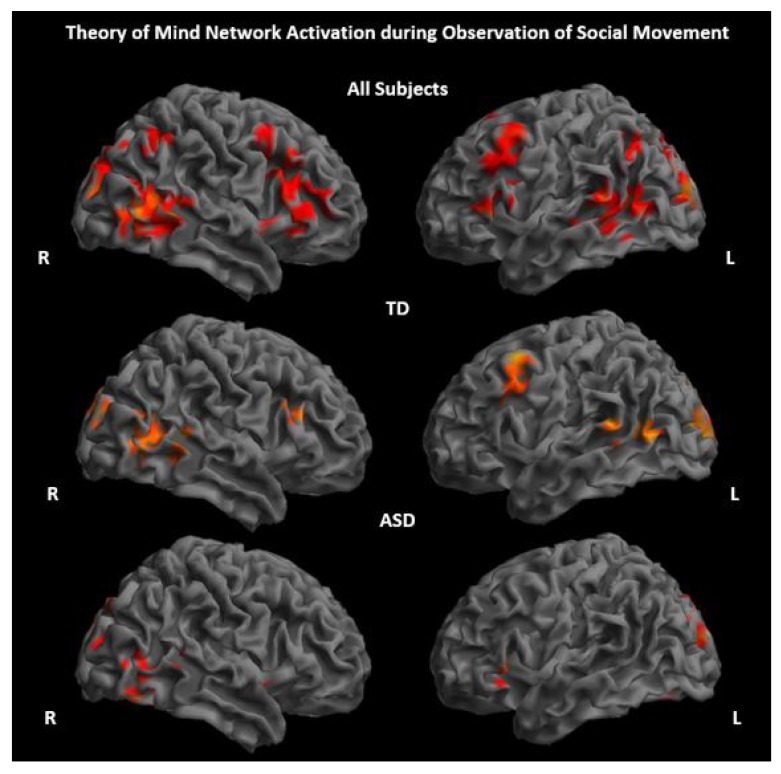
Within group activation results for social movement over fixation showing activation in classic ToM regions. All results significant at p<.005, 80 voxel threshold.

ASD and TD groups showed significantly increased activity primarily in regions considered part of the ToM network, including the pSTS, TPJ, and MPFC, while observing socially meaningful movements of shapes and characters compared to a fixation baseline (Fig. **2** and Supplementary Material **S1**). There were no significant between-group differences for the contrast social movement > fixation. All activation results reported are significant at p<.005, 80 voxel threshold.

**Fig. (3):**
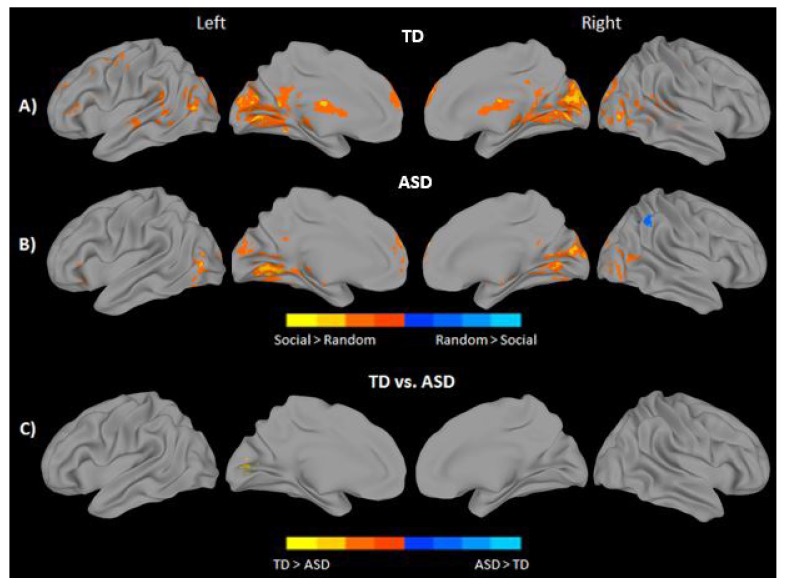
Within group results for **A)** typically developing (TD) individuals and **B)** individuals with autism spectrum disorder (ASD) and **C)** significant between group differences for the contrast of social vs. random movement. All results are significant at a Monte Carlo corrected threshold of p<.005, 80 voxel extent. Warm colors represent greater activation in the social movement condition compared to the random movement condition. No areas demonstrated greater activity for the random condition.

Observing social movement in contrast to random, non-goal directed movement, regardless of character, elicited robust bilateral activation in areas such as the calcarine gyrus, lingual gyrus, and cuneus in both groups. Bilateral activation of MPFC was also seen for both groups, as well as activation in LIFG. The TD group further recruited bilateral precuneus and superior and middle temporal regions to a greater extent when observing social compared to random movement. No brain areas showed significantly greater response when observing random movement in the TD group. In contrast, the ASD group showed greater activation in the Inferior Parietal Lobule (IPL) when observing random movement of objects (Figs. **3A**, **3B**, and Supplementary Material **S2**). Direct statistical comparison between groups revealed significantly reduced activation of the left lingual and calcarine gyri to social movement in the ASD group (Fig. **3C** and Supplementary Material **S2**). No inverse effects were found for this contrast (ASD > TD).

#### 3.3.2 Human Figures

**Fig. (4):**
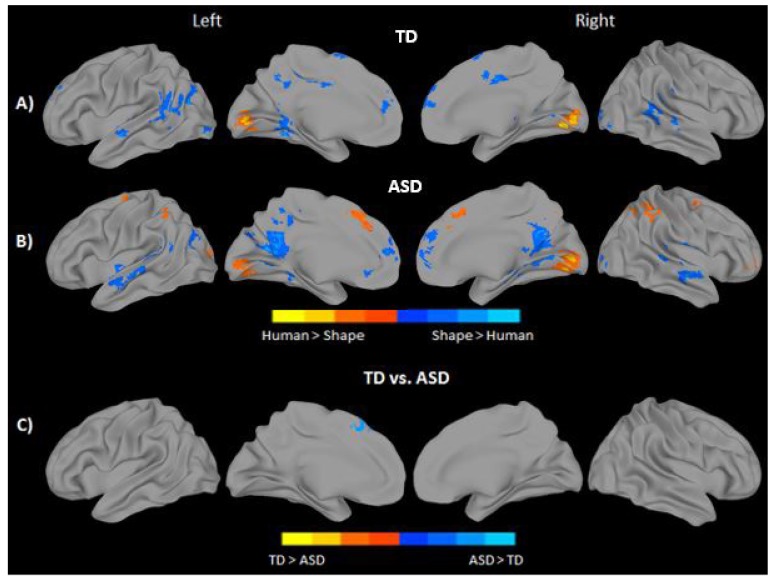
Within group results for **A)** Typically Developing (TD) individuals and **B)** individuals with Autism Spectrum Disorder (ASD) and **C)** significant between group differences for the contrast of human vs. shape character. All results are significant at a Monte Carlo corrected threshold of p<.005, 80 voxel extent. Warm colors represent greater activation in response to the human character compared to the shape character, while cool colors represent greater activation during the shape character condition.

Observing human-like stick figure characters in contrast to shapes, regardless of their movement type, revealed greater bilateral calcarine and lingual gyrus activation in both groups. The ASD group additionally activated the IPL, and supplemental motor/medial frontal areas bilaterally in response to human like characters. In contrast, observing shapes elicited greater bilateral activation in medial and superior temporal regions, MPFC, middle and anterior cingulate, fusiform/parahippocampal areas, and visual areas including the inferior and middle occipital in both groups. The TD group additionally recruited bilateral SMA, middle cingulate, and left TPJ in response to shape characters over and above stick-figures. In contrast, the ASD group demonstrated additional activation in bilateral precuneus (see Figs. **4A**, **4B**, and Supplementary Material **S3**). Direct statistical comparison between groups revealed no areas of greater activity in the TD group when observing stick figures compared to the ASD participants. On the contrary, significantly greater activation in the left superior medial frontal gyrus, BA 8, was found for the ASD group when observing the stick figure character compared to their TD peers (Fig. **4C** and Supplementary Material **S3**).

### 3.4 Regression Analysis

**Fig. (5):**
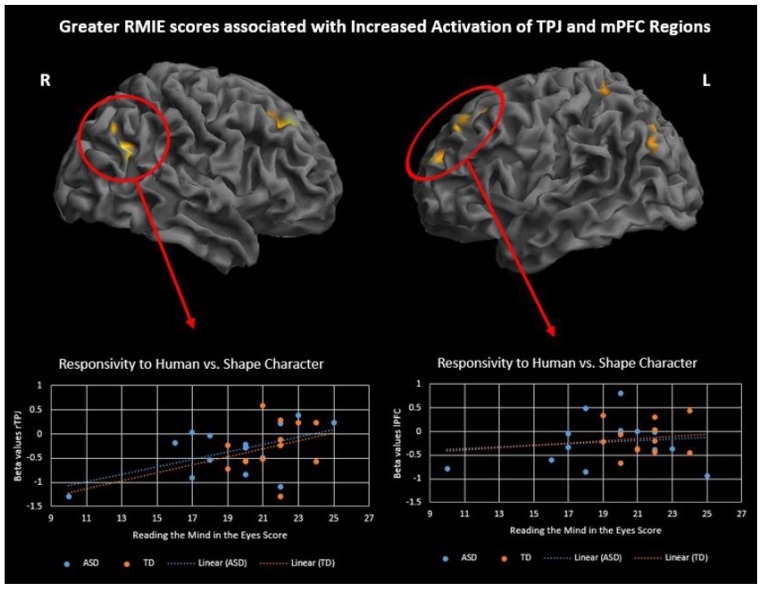
Positive correlation between RMIE scores and brain activity while observing human-like actors while controlling for IQ. Results significant at p<.001, 50 voxel threshold.

**Fig. (6):**
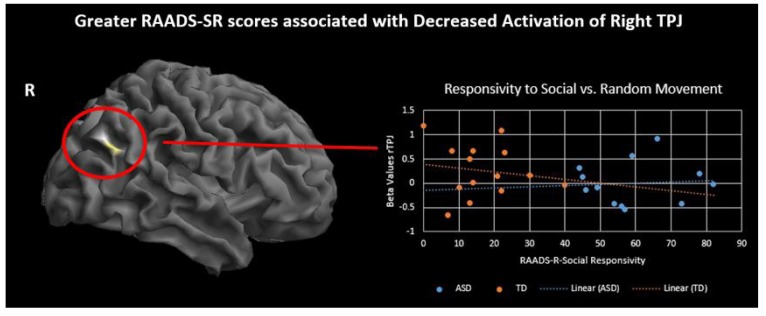
Negative correlation between RAADS-R-Social Responsivity scores and activation in the right TPJ during observation of social movement while controlling for IQ. Results significant at p<.001, 50 voxel threshold.

To investigate differences in brain activation related to ToM skills and ASD symptoms, performance on two behavioral measures, RMIE and RAADS-R social responsivity, was regressed on the activation results for two contrasts of interest, social > random movement and human > triangle character. Regression analysis was performed with all subjects while controlling for IQ. Results are significant at p<.001, 50 voxel threshold. There was a significant positive correlation between RMIE scores and activation in several social brain regions during observation of human character movement (Fig. **5** and Supplementary Material **S4**). As RMIE scores increased, activity in the bilateral TPJ and superior frontal gyri increased in response to watching human-like stick figure characters compared to shapes. Additionally, a significant negative correlation was found between rTPJ activity while observing social movement and RAADS-R social responsivity scores such that greater ASD symptoms were associated with less rTPJ response (Fig. **6** and Supplementary Material **S5**).

## 4 DISCUSSION

Contrary to our expectations, the anthropomorphism of the character did not influence attributions of agency at the behavioral level in either the ASD or TD group. This may be due to the rather rudimentary nature of stick figures as anthropomorphic agents. It could also be a reflection of our high functioning sample of ASD participants or the relatively low difficulty level of the task given that participants only had to label the action as social rather than describing the specific intentions of the characters. On the other hand, it may imply that the specific aspects of an agent, whether social or not, do not influence social attribution. However, it should be noted that the anthropomorphic features did influence brain activation. The observation of geometrical shapes, not the more anthropomorphic stick figures, recruited greater ToM network activation. Interestingly, geometrical shapes activated regions associated with ToM processing (superior and middle temporal regions, precuneus, and MPFC) over and above that seen for stick figures. It is possible that, without the inherent social/biological cue present in the stick figure character, additional ToM resources were needed to accurately process the information and attribute agency. On the other hand, this could reflect very early emergence of an “uncanny valley” situation in that less explicitly anthropomorphic characters are deemed more natural when engaged in social movements. In contrast to shapes, stick figures strongly activated lower order visual regions likely reflecting the more complex visual aspects of the stimuli, *i.e.* lines and orientation.

Group difference analysis showed one region within the dorsomedial PFC was more active when viewing human characters in ASD than in TD. Dorsomedial PFC has been found to be consistently recruited when thinking about the mental states of others [41] and making self-other distinctions [42]. MPFC has frequently been reported as underactive during social attribution tasks using only shapes in ASD [25-27]. An increase in dmPFC activity was found selectively for the human character relative to TD peers in our study. It is possible that individuals with ASD required the additional social prime of a stick figure to fully engage the ToM network while TD individuals did so more implicitly, irrespective of character. Outside of mentalizing, the dmPFC is also found to be involved in predicting and making decisions under uncertainty [43, 44]. Activity in BA8 specifically has been shown to correlate with rising uncertainty and is sensitive to both internal and external causes of uncertainty [44]. Increased activation in this area relative to shapes in the ASD group may reflect greater ambiguity when deciding intent of a character that more closely resembles a human. Greater uncertainty triggered by the social actor in the ASD group may explain why mPFC activity, previously shown to be decreased in ASD during ToM tasks, was increased in this study.

As expected, observation of socially oriented movement activated the ToM network broadly. Unlike the majority of previously reported [25-27, 45, 46], but not all [47], studies, there was no group difference in ToM network activation between ASD and TD groups. In many of these studies, participants were required to identify the specific mental state of the characters (*e.g*., deceiving, coaxing); whereas our task simply asked them to classify the movement as social or random. This made it a substantially easier task and is likely the main contributor to the lack of group differences. High functioning adults with ASD are often able to pass higher level ToM tasks, only demonstrating difficulties in real world scenarios or with subtler aspects of social cognition. Eliminating the need to infer the specific intentions of the characters may have reduced the complexity of the task enough so as not to produce group differences. It is also likely that low statistical power due to smaller sample size and aspects of the experimental design may have obfuscated other true differences. On the other hand, this could suggest that, at least in very high functioning populations, individuals with ASD recruit ToM resources to the same extent as their TD peers when making social attributions. Outside of ToM network, TD individuals had greater activation of lower order visual regions to socially oriented movement than their ASD counterparts had. Visual motion perception for both social and nonsocial tasks has been shown to be impaired in ASD including primary visual processing of biological motion [34, 48, 49]. Our results suggest this primary visual processing deficit, at least in the context of intentional attribution, is selective to social movements. As such, it could reflect aberrations occurring early in primary sensory areas leading to difficulties with more complex, downstream processing such as agency detection and mentalizing.

ToM ability, rather than being an all or nothing skill, varies among individuals and neural correlates of this variability have been documented on the RMIE task [50, 51]. We report that greater bilateral TPJ activation while observing human-like characters is correlated with higher ToM skill across groups. Furthermore, right TPJ activity for social movement was negatively correlated with ASD symptoms of social responsivity indicating less activity in this region for those with more severe symptoms. Previous research found a similar result when making mental and physical judgements about self and others [52]. In their study, rTPJ was underactive for the mentalizing condition and this activity predicted poorer real-world social functioning. TPJ has been considered as a crucial social brain hub as well as an integration center for various sensory modalities and plays an important role in attentional orienting [53, 54]. Converging evidence that all of these processes may be impacted in ASD, and the TPJ’s ability to predict real-world social functioning, makes this an important region of study in ASD.

There are a few limitations to the current study that must be considered when evaluating these results and understanding their implications. First, exclusion of participants based on movement or poor understanding of the task further reduced the final sample size to 14 participants per group, which is relatively smaller. This would have limited the statistical power to detect true effects. Second, aspects of the experimental design may have also contributed to limited power due to infrequent repeats of task conditions. Each participant viewed 5 video clippings related to each of the 4 conditions resulting in 92 to 117 seconds of task based data per condition. This may not have been sufficient to detect all true effects and may have contributed to some of the null findings of this study. Lastly, as in all fMRI studies, the nature of the scanning environment and task demands required that our ASD sample be rather high functioning, able to remain still in an enclosed space for an hour, and not possess any sensory aversions severe enough to preclude being in a noisy environment. As such, our final ASD sample may not reflect the ASD population at large and may limit generalizability to these findings.

## CONCLUSION

Overall, the results of the present study contribute to a greater understanding of the contextual features of social attribution in typical development and in adults with ASD. Anthropomorphic cueing may enable individuals with ASD to fully engage ToM network during intentional attribution. Further, reduced primary visual processing of dynamic social movement may point to an upstream contributor to downstream ToM deficits. Lastly, TPJ activity may provide a mechanism for individual differences in ToM abilities broadly and relate to real world social deficits. Future research should consider investigating how the degree of anthropomorphism influences attributions using life-like stimuli on a continuum of anthropomorphic stimuli.

## Figures and Tables

**Table 1:** Demographic information for the study sample. Values are presented as mean (standard deviation; range). T statistics and p values are for independent sample t-tests between group means.

***Groups***				
	***ASD (n=14)***	***TD (n=14)***	***t***	***p***
**Gender**	10 M; 4 F	13 M; 1 F	-	-
**Handedness**	13 R; 1 L	13 R; 1 Ambidextrous	-	-
**Age (years)**	25.07 (±6.16; 17-40)	24.86 (±5.35; 19-36)	0.10	0.92
**Full Scale IQ (WASI)**	117.57 (±12.97; 99-140)	117.23 (±8.78; 105-134)	0.08	0.94
**Verbal IQ**	117.07 (±11.16; 101-135)	112.38 (±11.12; 92-129)	1.09	0.29
**Performance IQ**	114.00 (±15.81; 89-138)	118.54 (±8.17; 103-132)	0.93	0.36
**RAADS-R total**	131.29 (±32.19; 72-181)	42.71 (±23.57; 3-78)	8.31	<.001
**Mind in the Eyes**	19.21 (±3.68; 10-25)	21.46 (±1.66; 19-24)	2.02	0.05

**Table 2:** Behavioral data from the in-scanner task. M = mean, SD = standard deviation. T statistics and p values are for independent sample t-tests between group means.

**Condition**	***M_ASD_***	***SD_ASD_***	***M_TD_***	***SD_TD_***	***t***	*p*
**Accuracy (%)**						
Social Shape	78%	1.6	77%	1.8	0.83	0.83
Random Shape	71%	2.3	71%	2.1	0.00	1.00
Social Human	70%	1.8	63%	1.7	1.09	0.28
Random Human	81%	2.0	80%	1.9	0.19	0.84
**Reaction Time (*msec*)**						
Social Shape	4141.97	1561.75	4102.83	1274.78	0.07	0.94
Random Shape	3531.35	1275.78	3633.17	1386.61	0.20	0.84
Social Human	3029.04	1086.87	3438.87	973.31	1.05	0.30
Random Human	3845.35	1282.20	4164.76	934.35	0.75	0.45
