# Supplementary material for: Brain Responses Underlying Anthropomorphism, Agency, and Social Attribution in Autism Spectrum Disorder
Source: Open Neuroimag J. 2018 Mar 30;12:16–29. doi: 10.2174/1874440001812010016 (PMC5885471; doi:10.2174/1874440001812010016)
Supplement: Supplementary file 1 [file TONIJ-12-16_SD1.pdf]

Send Orders for Reprints to reprints@benthamscience.ae

The Open Neuroimaging Journal, 2018, *12*, i-iii

BENTHAM OPEN

CrossMark

The Open Neuroimaging Journal

Supplementary Material

Content list available at: www.benthamopen.com/TONIJ/

DOI: 10.2174/1874440001812010016

Brain Responses Underlying Anthropomorphism, Agency, and Social Attribution in Autism Spectrum Disorder

Carla J. Ammons, Constance F. Doss, David Bala and Rajesh K. Kana*

Department of Psychology, University of Alabama at Birmingham, Birmingham, AL, USA

Received: November 28, 2017 Revised: February 27, 2018 Accepted: March 03, 2018

SUPPLEMENTARY MATERIALS

Table 1. Brain regions, cluster size, MNI coordinates, and t-scores for significant activation clusters in the Social - Fixation condition.

Region	Hem.	*k*	MNI			*t*
Within Group Results<br>*Social > Fixation*<br>*TD*						
Lingual/Calcarine	L./R.	3712	14	-88	16	7.60
Middle/Superior Temporal	R.	846	60	-40	12	5.61
Middle Frontal Gyrus	L.	420	-38	14	52	5.77
Lingual	R.	235	26	-30	-2	5.80
Middle Temporal Gyrus	L.	215	-36	-58	8	5.99
Inferior Frontal (Tri)	R.	214	56	26	22	6.23
Middle/Superior Temporal	L.	198	-56	-42	12	5.60
Superior Medial Frontal	L./R.	175	-2	38	46	4.08
Hippocampus	L.	148	-26	-34	-2	5.80
ASD						
Lingual/Calcarine	L./R.	6797	20	-64	-10	10.16
Putamen	L.	311	-24	8	0	6.43
Hippocampus	L.	250	-28	-22	-8	7.09
Inferior Frontal (Tri)	L.	155	-54	18	2	5.31
Middle/Superior Temporal	R.	125	62	-46	8	4.51

Note. L, left; R, right; Hem, hemisphere; *k*, cluster size defined as the number of voxels.

Table 2.Brain regions, cluster size, MNI coordinates, and t-scores for significant activation clusters in the Social - Random condition.

Region	Hem.	*k*	MNI			*t*
Within Group Results<br>*Social > Random*<br>*TD*						
Lingual/Calcarine	L./R.	6238	12	-90	16	9.25

(Table 2) contd.....

Region	Hem.	k	MNI			t
Caudate	L./R.	1433	-20	-32	2	4.50
Superior Medial Frontal	L./R.	618	6	58	34	7.26
Middle Temporal Gyrus	R.	379	44	-76	0	4.11
Middle Temporal Gyrus	L.	369	-46	-66	-20	5.70
Superior Temporal Gyrus	L.	253	-56	-42	18	5.02
Precentral Gyrus	L.	114	-46	12	46	4.92
Middle Temporal Gyrus	L.	86	-52	-22	0	4.54
ASD						
Lingual/Calcarine	L./R.	3717	-20	-62	-8	8.09
Middle Occipital Gyrus	L.	206	-44	-76	4	9.24
Putamen	R.	189	30	4	-6	5.19
Superior Medial Frontal	L./R.	158	4	58	24	7.15
Fusiform Gyrus	L.	131	-40	-52	-20	5.87
Random > Social						
ASD						
Inferior Parietal Lobule	R.	155	44	-52	38	4.87
Between Group Results<br>*Social > Random*<br>*TD>ASD*						
Calcarine Sulcus	L.	97	-6	82	8	3.84

Note. L, left; R, right; Hem, hemisphere; *k*, cluster size defined as the number of voxels.

Table 3. Brain regions, cluster size, MNI coordinates, and t-scores for significant activation clusters in the Human - Shape condition.

Region	Hem.	k	MNI			t
Within Group Results<br>*Human > Shape*<br>*TD*						
Lingual/Calcarine	L./R.	791	12	-72	-6	8.26
ASD						
Lingual/Calcarine	L./R.	1321	10	-78	-2	9.79
Inferior Parietal Lobule	R.	508	44	-52	50	8.03
Superior Medial Frontal	L./R.	254	0	22	48	5.19
Inferior Parietal Lobule	L.	166	-44	-52	46	3.95
Precentral Gyrus	L.	144	-16	-20	56	5.78
Precuneus	R.	102	16	-52	48	6.22
Shape > Human						
TD						
Middle Temporal	L.	563	-40	-68	20	4.96
Lingual Gyrus	L.	455	-30	-48	-12	5.50
Middle/Superior Temporal	R.	300	52	-52	6	5.13
Inferior Occipital	R.	199	24	-90	-6	6.12
Cingulum	L./R.	195	4	-8	40	4.56
Superior Medial Frontal	R.	153	6	60	20	4.40
Superior Temporal	R.	107	56	-24	2	4.60
Superior Medial Frontal	L.	105	-10	46	16	4.06
Supplementary Motor Area	L./R.	91	8	8	68	4.50
ASD						
Precuneus	L./R.	2060	-16	-60	14	11.45
Superior/Middle Temporal	R.	330	54	-14	-12	7.57
Superior/Middle Temporal	L.	240	-52	-4	-14	4.21
Superior Temporal	R.	180	52	-34	6	8.31
Inferior Frontal Gyrus	R.	159	20	-90	-8	4.46
Superior Medial Frontal	L.	104	-10	50	12	5.78

(Table 3) contd.....

**Region**	**Hem.**	***k***	**MNI**			***t***
Middle Occipital	L.	118	-44	-74	22	4.12
Superior Medial Frontal	R.	92	6	60	8	4.40
Superior Temporal Gyrus	R.	81	62	-16	8	4.32
Between Group Results<br>*Human > Shape*<br>*ASD > TD*						
Dorsomedial Prefrontal	L.	106	-6	30	52	3.90

Note. L, left; R, right; Hem, hemisphere; *k*, cluster size defined as the number of voxels.

Table 4. Brain regions, cluster size, MNI coordinates, and t-scores for significant activation clusters in the Human – Shape condition positively correlated with RMIE scores, controlling for IQ.

**Region**	**Hem.**	***k***	**MNI**			***t***
All subjects<br>*Human > Shape*						
Cingulum	L./R.	393	-10	-40	40	7.20
Superior Medial Frontal	L.	328	-16	20	42	6.77
Superior Medial Frontal	R.	281	24	26	42	6.56
Precuneus	L.	184	-2	-66	34	4.33
Putamen	R.	173	32	-10	2	4.97
Precuneus	L.	167	-2	-44	62	5.38
Angular Gyrus	R.	137	467	-60	26	6.02
Superior Medial Frontal	L.	106	-26	50	30	5.59
Mid Cingulum	R.	84	6	-10	38	5.49
Mid Cingulum	L.	83	-8	0	36	4.96
Caudate	R.	74	8	24	-4	5.25
Postcentral Gyrus	R.	69	26	-36	62	4.70
Inf Parietal/Mid Occipital	L.	60	-38	-74	30	4.08

Note. RMIE, Reading the Mind in the Eyes; L, left; R, right; Hem, hemisphere; *k*, cluster size defined as the number of voxels.

Table 5. Brain regions, cluster size, MNI coordinates, and t-scores for significant activation clusters in the Social-Random condition negatively correlated with RAADS-R-SR scores, controlling for IQ.

**Region**	**Hem.**	***k***	**MNI**			***t***
All subjects						
Social > Random						
Temporal Parietal (TPJ)	R.	88	44	-70	34	4.59

Note: RAADS-S-SR, Ritvo Autism Asperger Diagnostic Scale Revised – Social Responsivity Subscale; L, left; R, right; Hem, hemisphere; *k*, cluster size defined as the number of voxels.

© 2018 Ammons *et al.*

This is an open access article distributed under the terms of the Creative Commons Attribution 4.0 International Public License (CC-BY 4.0), a copy of which is available at: https://creativecommons.org/licenses/by/4.0/legalcode. This license permits unrestricted use, distribution, and reproduction in any medium, provided the original author and source are credited.
